# Placental lesions in birth asphyxia and hypoxic ischemic syndrome

**DOI:** 10.3892/mi.2024.205

**Published:** 2024-11-18

**Authors:** Andreea Calomfirescu-Avramescu, Luminiţa Ceauşelu, Mihaela Demetrian, Vlad Dima, Anca Bălănescu, Paul Bălănescu, Andrada Mirea, Adrian-Ioan Toma, Sorina Crenguța Șerboiu, Oana Maria Pătrașcu, Isam Al Jashi, Ioan Gherghina

**Affiliations:** 1 Filantropia Clinical Hospital, 011132 Bucharest, Romania; 2 Department of Pediatrics, Carol Davila University of Medicine and Pharmacy, 050474 Bucharest, Romania; 3 Department of Neonatology, Faculty of Medicine, Titu Maiorescu University, 040441 Bucharest, Romania; 4 Alessandrescu-Rusescu National Institute for Mother and Child Health, 20382 Bucharest, Romania

**Keywords:** birth asphyxia, hypoxic ischemic lesions, placental lesions, Amsterdam Criteria

## Abstract

Birth asphyxia is a severe condition that includes a number of potential pathways of occurrence both *in utero* and during childbirth. The present study aimed to identify and describe specific macroscopic and microscopic placental injuries in birth asphyxia to serve as an effective tool to stratify the potential further evolution of a newborn, as hypoxic ischemic encephalopathy can be responsible for neonatal death or severe neurological sequelae further, compromising the quality of life of the affected individual. For this purpose, an observational prospective study was conducted over a period of 3 years. A total of 62 patients diagnosed with birth asphyxia, who had a placental histopathological examination performed were enrolled in the study. The control group consisted of 69 term newborns that required neonatal intensive care for at least 3 days, in the same time period, for any other reason and that also had available placental examinations. In the present study, placental histopathological lesions identified in birth asphyxia have been classified according to the Amsterdam Criteria. Data gathered from both groups were analyzed by applying specific statistical tests for each type of variable and hypothesis. Thus, umbilical cord abnormalities were associated with hypoxic ischemic encephalopathy in a statistically significant manner when comparing the birth asphyxia group of newborns with the control group. In addition, a high statistical level of significance was identified for microscopical lesions, such as maternal and fetal vascular malperfusion and the occurrence of hypoxic ischemic syndrome when comparing the two groups (P=0.01). The macroscopic and microscopic placental examination can provide critical information for the evolution of the disease in selected newborns according to the identified lesions.

## Introduction

Birth asphyxia and hypoxic ischemic syndrome a very consequential illnesses that are linked to elevated rates of infant mortality and significant neurological consequences (1). The syndrome is a topic of contention in scientific literature, with numerous writers attempting to enhance its definition throughout time, resulting in increased precision, but also in increased complexity. The prevalence of neonatal asphyxia has experienced a significant increase in recent years, including a spectrum of severity from moderate to severe manifestations. Consequently, it has emerged as a fundamental challenge encountered within neonatology units (2). The phrase ‘birth asphyxia’ is employed to describe a situation in which a baby exhibits poor Apgar scores, notable metabolic acidosis detected in umbilical cord blood samples, and a modified neurological examination (with or without EEG correspondence) in the absence of any indications of other etiologies of encephalopathy (3).

Asphyxia during the perinatal period leads to the formation of hypoxic cerebral lesions, which have a marked impact on the cognitive, neurological and motor development of neonates. Defining the etiology of hypoxia is crucial for effectively treating the cause and achieving a positive outcome.

The etiology of hypoxic ischemic syndrome at birth is multifactorial, with both intrapartum and antenatal factors. When affected, each link of the pathological chain causes the onset of a mild or more severe form, thereby the need to investigate the most accessible parts of the process. The diagnosis of hypoxic ischemic encephalopathy is made on clinical criteria (Sarnat classification), as well as on laboratory tests (evidence of metabolic acidosis, multiple organ damage) and brain imaging (EEG and MRI) (4). The treatment of hypoxic ischemic encephalopathy should be prompt to overcome the onset of permanent neurological sequelae. It has been extensively studied over the last decade, leading to the identification of extensive neuroprotective strategies, such as controlled hypothermia or the use of several molecules, such as erythropoietin and other molecules still under investigation (5).

The identification of pathogenic processes occurring during gestation has been demonstrated by the study of placental pathology. The prompt identification of the hypoxic-ischemic lesion is crucial in the context of encephalopathy to facilitate timely intervention. Despite extensive research conducted on maternal risk factors and birth events associated with prenatal hypoxia or cerebral palsy, there are a limited number of studies available that have established direct associations between placental histological signs and neurological impairment (6).

The histological analysis of the placenta is often conducted primarily in instances of challenging births, to identify distinct alterations that are linked to a higher incidence of infant mortality. In recent years, there has been a growing scientific interest in investigating the association between the diagnosis of hypoxic ischemic encephalopathy and abnormalities in the placenta. The Amsterdam Placental Workshop Group Criteria were formulated in 2016 to establish a uniform categorization system for placental lesions (7).

Perinatal asphyxia is linked to placental abnormalities that affect blood flow to the fetus. The identified lesions on the umbilical cord included issues, such as damaged velamentous vessels, cord rupture, hypercoiled chords and cord hematoma. Additionally, there were instances of chorioamnionitis accompanied by fetal vasculitis and fetal vascular malperfusion (8,9).

In the present study, it was hypothesized that by carefully examining a single ‘key’ component, such as the placenta, the identification of etiological associations between placental abnormalities and the occurrence of birth asphyxia would be possible. In order to provide a rapid tool for stratifying the probable future evolution of newborns, the present study aimed to identify and characterize particular macroscopic and microscopic placental damage in hypoxic neonates.

## Subjects and methods

### Subject information

An observational, prospective, non-interventional study was undertaken at the ‘Filantropia’ Clinical Hospital in Bucharest, Romania, spanning a duration of 3 years from 2020 to 2022. The research was conducted with the requisite authorization from the Ethics Council of ‘Filantropia’ Clinical Hospital and according to the privacy protocols established for the participating patients. Prior to the inclusion of the mother and newborn in the study, the parents or legal guardians provided their signature on an informed consent agreement. The research was carried out following the principles outlined in the Declaration of Helsinki on Human Rights.

Relevant maternal information, including the social, medical and family history of the mother, was gathered in conjunction with data about the newborn. These data encompassed various aspects, such as fetal heartbeat during labor, gestational age, delivery type, birth weight and sex. Additionally, data from the clinical examination of the newborns, including changes associated with perinatal hypoxia and Apgar score, were also provided. In addition, laboratory findings were documented, including hemograms, metabolic parameters, pH and acid-base balance parameters, all of which were collected in a dynamic manner. However, the database was updated with placental histopathology examination data, including both micro- and macroscopic images.

### Examination procedures

The investigation involved a macroscopic examination of the placenta, umbilical cord and membranes. The macroscopic lesions were from predefined categories: In the case of the placenta, weight and appearance; in the case of the umbilical cord, length and appearance, insertion; and in the case of the membranes, color and appearance. The weight of the placenta was measured within the initial hour following delivery, after the removal of the membranes and umbilical cord. The assessment of the umbilical cord encompassed various aspects, such as its length, the location of its insertion about the center or borders of the placenta, the existence or absence of a hypercoiled (≥3 coils per 10 cm) or hypocoiled (1 coil per 10 cm) look, and the identification of a single umbilical artery. The membranes were described as having an opaque look and colour and were inserted in a circumvallate or circummarginal manner.

An experienced pathologist conducted the placental examination. On fresh placenta sections, the microscopic examination was performed before they were embedded in paraffin and stained with hematoxylin and eosin (with Mayer's hematoxylin solution, supplied by Merck, for a duration of 15 min at room temperature). A total of five samples (1.5-2-cm-thick) were obtained for microscopic examination: One from the membranes, one from the umbilical cord and three from the placental parenchyma. In the cases in which the placenta examination could not be conducted immediately after birth, the samples were refrigerated at 4˚C, for a maxim period of 24 h. During the examination, the microscope used was Zeiss Axioscope 5 and the images were captured with Zeiss Axiocam 208 (Zeiss AG). The classification included the primary six pre-established classifications of injuries, according to the Amsterdam Criteria: Maternal vascular malperfusion, fetal vascular malperfusion, chronic villitis of unknown etiology, delayed villous maturation, chorioamnionitis and abruption. The following abnormalities were associated with maternal vascular malperfusion: Placental hypoplasia (weight below the 10th percentile for gestational age), villi abnormalities (hypoplasia, increased fibrin deposits at villous levels, necrosis) and placental infarction.

Fetal vascular malperfusion includes both segmental and generalized alterations, which can appear as thrombosis with or without occlusive thrombi, avascular villi, intramural fibrin deposits in the major veins and stromal-vascular karyorrhexis. Chronic villitis of unknown etiology that associates vascular obstruction, and avascular villi was diagnosed as the presence of inflammation that affects >30% of the distal villi. Delayed villous maturation is represented by villous maturation inappropriately immature for gestational age across the peripheral villi, which includes at least 30% of a section. The defining characteristic of chorioamnionitis is the presence of inflammatory cells inside the layers of chorion membranes. This condition is sometimes accompanied by necrosis and acute vasculitis at certain levels of the umbilical cord and chorion. The clinical manifestation of abruptio placentae was characterized by the observation of retroplacental hemorrhage. Placentas with distinct lesions across the six categories were excluded from the analysis. The Zeiss Axioscope 5 microscope (Zeiss AG) was used to analyze all placental samples.

### Study groups and criteria

**Figure 1:**
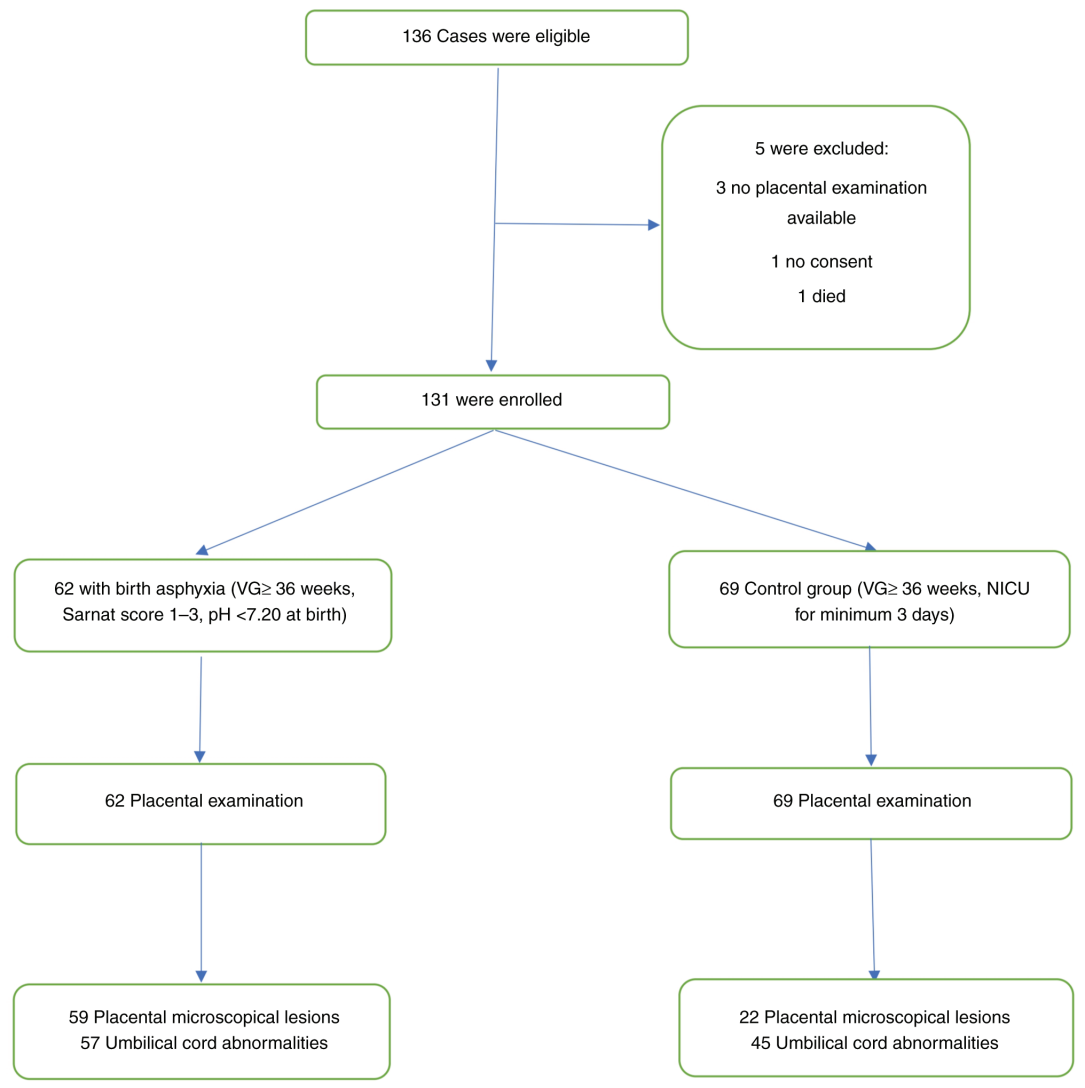
Consort diagram of the study selection process.

The study group included 62 newborns who had perinatal asphyxia, with a gestational age >36 weeks and a diagnosis of hypoxia at birth (mild, moderate and severe). The exclusion criteria were the following: Patients who had viral intrauterine infections, congenital abnormalities, chromosomal anomalies and twin pregnancies. The control group consisted of 69 newborns with a gestational age >36 weeks who required neonatal intensive care for a minimum of 3 days for any other pathology, excluding hypoxia. The exclusion criteria for the control group were the following: Congenital abnormalities, chromosomal anomalies or were unable to undergo placental examination. Placental examinations were conducted in all patients. The diagnosis of birth asphyxia in newborns was established with a comprehensive neurological clinical examination and subsequently categorized using the Sarnat score, which ranged from 1 (mild) to 3 (severe). The blood collected from the clamped umbilical cord immediately after birth had a pH value <7.20. A complete acid-base study was conducted on the collected blood samples, including measurements of pH, pCO_2_, pO_2_ and base deficit. Patients who were excluded from the study did not match the specified inclusion criteria. All data are summarized in Fig. 1.

### Statistical analysis

SPSS 25.0 for Windows (IBM Corp.) was used to statistically analyze the recorded data. The Chi-squared test or an unpaired t-test were employed for independent sample analyses, whereas Mann-Whitney tests were used to analyze non-parametric variables. Fisher's test was also applied for each variable that had a count of ≤5. A value of P<0.05 was considered to indicate a statistically significant difference.

## Results

A total of 136 patients who met the inclusion criteria were enrolled; 5 patients were excluded. In addition, 36 (27.48%) of the patients were males. The incidence of cesarean section was found to be 28.24%, with a total of 37 neonates being born with emergency cesarean section.

Cardiotocography (CTG) was used to continuously monitor the mothers during labor. The recorded changes included fetal bradycardia, fetal tachycardia and variability. Of note, 60.3% (78 pregnancies) exhibited CTG abnormalities, with 51 pregnancies (82.3%) in the asphyxia group (P=0.01) and 27 pregnancies (39.1%) in the control group. Tachycardia was observed in 18 cases (29.0%) (P=0.01) and bradycardia (P=0.01) in 23 neonates (37.1%) with asphyxia. In addition, lower variability was observed in 10 neonates (16.1%) with hypoxia (P=NS). Within the control group, 12 cases (17.4%) experienced fetal bradycardia, 7 cases (10.1%) experienced fetal tachycardia, and 8 cases (11.6%) exhibited lower variability.

Complicated pregnancies accounted for a substantial portion of the cases, specifically 92 (70.2%). Among the 62 neonates with asphyxia, 56 (90.3%) were born from pregnancies with complications (P=0.01), whereas in the control group, only 36 (52.2%) had problems. Out of the numerous medical conditions related to pregnancy, 25 cases (19.0%) were attributed to pregnancy-induced hypertension, with 18 cases (29.0%) occurring within the study group (P=0.01). Gestational diabetes was present in 33 (25.2%) cases. Among these, 16 (25.8%) neonates had asphyxia (P=NS), while 17 (24.6%) were in the control group. In addition, there were 14 cases (10.7%) of preeclampsia; in the study group, there were 11 (17.7%) cases (P=0.02), while in the control group, there were 3 (4.3%). Additionally, pregnancy cholestasis was present in 20 cases (15.3%), 11 (17.7%) with asphyxia (P=NS), and 9 (13.0%) in the control group. In addition, 5 (3.8%) patients were under psychiatric treatment at the time of birth, 4 (6.4%) from the study group (P=NS), and 1 (1.4%) from the control group. The features of the patients are presented in Table I.

Additionally, it is important to note that the control group consisted of neonates who had been diagnosed with transient tachypnea of the newborn 27 (38%), early neonatal sepsis 15 (23%), hypoglycemia 14 (20%), pneumonia 9 (14%) and meconium aspiration syndrome 4 (5%).

Newborns with perinatal asphyxia exhibited a lower umbilical cord pH value, measuring 7.05 (P=0.01). Additionally, they had a smaller umbilical cord base deficit of -13.4 mmol/l (P=0.01), a higher umbilical cord pCO_2_ of 64.2 mmHg (P=0.01) and greater median ventilation days (P=0.01). It is important to note that the duration of hospital stay was significantly longer in individuals with asphyxia compared to the control group, with a P-value of.03 (Table I).

The macroscopic examination records of the placenta contained a number of indicators, such as placental weight, membrane aspect, appearance and implantation of the umbilical cord. The birth asphyxia group exhibited a significantly increased placental weight compared to the control group (P=0.01). Furthermore, 57 neonates (91.9%) with hypoxia exhibited umbilical cord anomalies, including: Umbilical knot, 10 neonates (16.1%); unique umbilical artery, 23 neonates (37.1%); abnormal insertion, 18 neonates (29%); hypercoiled, 4 neonates (6.5%); and hypocoiled, 2 neonates (3.2%). Furthermore, the occurrence of perinatal hypoxia was found to be linked with alterations in the umbilical cord, namely the presence of knots (P=0.01) and the existence of a distinct umbilical artery (P=0.01). These data are presented in Table II.

**Figure 2:**
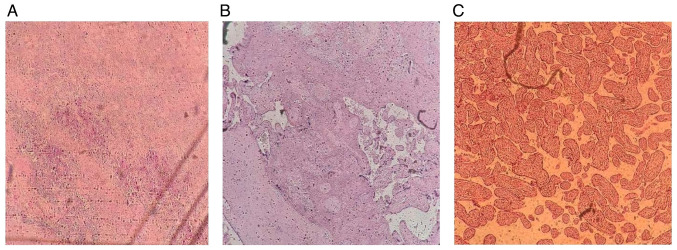
(A) Chorioamnionitis. (B) Villous fibrinoid deposition and necrosis. (C) Villous hypoplasia.

Placental microscopic changes were observed in 81 (61.8%) of cases, while 50 (38.2%) were normal. The most commonly identified lesions in birth asphyxia were chronic from the maternal vascular malperfusion [15 (24.2%)] and fetal vascular malperfusion [11 (17.7%)] class. In the control group, the normal aspect was predominant. Furthermore, the group with perinatal asphyxia exhibited a higher prevalence of chronic villitis of unknown causes, with 10 cases (16.2%) out of the 62 cases, compared with the control group with 3 cases (4.2%) out of the 69 cases. In instances of delayed villous maturation, similar findings were observed, with 12 cases (19.4%) out of 62 and 4 cases (5.6%) out of 69 cases. These results highlight a statistical association between these lesions and the occurrence of hypoxic lesions at birth, chronic villitis of unknown cause (P=0.01), and delayed villous maturation (P=0.01) (Table III). Some of the identified lesions are illustrated in Fig. 2.

Out of the total 7 cases of abruption, 6 (9.6%) cases were assigned to the study group, while 1 (1.4%) case was assigned to the control group. Due to its small number, the data did not demonstrate a strong significant statistical association between the two variables (P=0.05). In addition, there were no differences in the two groups regarding chorioamnionitis (P=NS). These data are presented in Table III.

A few other lesions were excluded during the placental histopathological examination as they could not be classified based on the criteria, and the placental histopathological examination (0.7%) did not reveal any chorangiosis. Moreover, in the instance of the presence of multiple simultaneous lesions [3 cases (2.2%)], they were classified according to the extent of the most extensive lesions (>30%).

The analysis of placental changes in relation to the severity of hypoxia revealed significant associations between maternal (P=0.01) or fetal (P=0.01) vascular malperfusion type lesions and severe perinatal asphyxia. In cases of moderate asphyxia, both fetal vascular malperfusion (P=0.01) lesions, and abruptio-type (P=0.02) lesions were observed. Additionally, the presence of chronic villitis of unknown etiology (P=0.01) and delayed villous maturation (P=0.01) type lesions were associated with mild perinatal asphyxia. Table IV provides a summary of all the statistical associations that have been found between the identified placental lesions and the degree of prenatal hypoxia.

## Discussion

The present study emphasized that a careful examination of both the umbilical cord and the placenta is critical to a better understanding of the multifactorial etiology of perinatal asphyxia. Based on the findings of the present study, the presence of both placental changes and the presence of hypoxic lesions at birth were associated with the presence of placental changes, as well as the umbilical cord.

The results highlight the importance of a placental examination in birth asphyxia, as also revealed by other studies, such as the study by Nielsen *et al* (10). Previous studies have revealed that both macroscopic and microscopic examinations are associated with hypoxic brain lesions (11-13). In the present study, the most common placental microscopic changes in perinatal asphyxia were from the maternal and fetal vascular malperfusion categories, unlike the control group where the majority of the placentas were normal. According to the classification of placental lesions, it can be concluded that the factors that determined the appearance of hypoxic lesions were chronic; these results were similar to those in the study by Bingham *et al* (14).

The results presented herein support the statement that hypoxic ischemic lesions are associated with both macro- and microscopic placental lesions and changes in the umbilical cord. As previously mentioned by Wintermark *et al* (9), the following pathological lesions of the umbilical cord were highlighted in the present study: Circumvallated membranes, single umbilical artery, hypo/hypercoiled and abnormal insertions. These lesions from the umbilical cord and membranes have been shown to be associated with fetal or perinatal mortality in previous studies (15-17). In the present study, they were associated with newborns with birth asphyxia (P=0.01).

The macroscopic examination of the placentas, as well as in other scientific studies, did not present significant changes (18). Lachapelle *et al* (19) demonstrated that the weight of the placenta and the ratio between birth weight and placenta proved to be a valuable factor. As observed in the present study, placental weight tended to be greater in newborns who had developing brain lesions, particularly in those with an extended lesion that caused hypoxic ischemic encephalopathy (P=0.01). The underlying mechanisms responsible for the augmentation of placental weight remain unknown. Placental hypertrophy during pregnancy may potentially serve as a protective mechanism for the fetus against stressful events *in utero*, which increases the risk of perinatal depression following hazardous events during labor and birth. This predisposition is primarily associated with the incidence of significant brain damage.

Other researchers have observed associations between the velamentous and marginal insertion of the umbilical cord into the placenta with the presence of hypoxic ischemic encephalopathy (20). These changes in the umbilical cord can cause acute injuries during labor or chronic circulatory obstruction, both of which can lead to birth asphyxia. Nomiyama *et al* (21) demonstrated the utility of Doppler imaging in the context of normal second-trimester sonography to identify cord insertion, particularly velamentous insertion. The rapid examination, characterized by a high level of precision, aids in the identification of pregnancies that are at risk. In the present study, the results were strongly associated with a single umbilical artery of the umbilical cord and birth asphyxia due to compromised blood flow through the umbilical vessels (P=0.01).

Redline (22) pointed out that placental lesions are the only explanation for asphyxia in some cases. Redline (22) identified the presence of placental pathological lesions in >90% of infants who subsequently developed cerebral palsy. The present study identified a statistically significant association between the presence of placental changes and the diagnosis of birth asphyxia (P=0.01). Redline (22) also demonstrated the association between placental vasculopathy and umbilical arterial pH under 7.10, which causes thrombotic events and mortality.

Due to the extensive lesions encountered in the histopathological examination, in the present study, the Amsterdam standardized classification was used to make the changes associated with hypoxic ischemic encephalopathy as specific as possible. Other classifying systems of placental injuries are used by other authors in their publications. For example, Chang *et al* (23) classified the lesions into inflammatory, vascular and other, while others have made more detailed classifications with 10 to 16 subgroups of histopathological lesions (20). In the present study, only six main groups were selected; the other lesions that could not be classified were very few and insignificant. Each lesion was categorized according to the Amsterdam Criteria (7) and included six large categories: Maternal vascular malperfusion, fetal vascular malperfusion, chorioamnionitis, chronic villitis of unknown aetiology, delayed villous maturation and abruption placenta. In the analyzed placentas, although several concomitant lesions were present, the division was performed after the dominant lesion from the pre-specific Amsterdam group.

Furthermore, in the present study, the most common microscopic placental lesions associated with the hypoxic-ischemic syndrome were in the category of maternal and fetal placental malperfusion, being labelled as chronic lesions predominant with the damage of villi (infarction, fibrin depositions and necrosis). Badawi *et al* (24), emphasized that fetal vascular malperfusion lesions present in newborns can cause both acute neonatal lesions and long-term damage through cord compression. Such lesions emphasize the multitude of ways of the etiology of hypoxic lesions. The results of the present study revealed an association between the presence of fetal vascular malperfusion-type lesions and the presence of perinatal asphyxia (P=0.01).

In addition, the present study identified a strong association between the presence of placental infarction and the occurrence of severe asphyxia, as in other research (25). The connection between placental vascular changes and the etiology of hypoxia at birth was a large research subject and has been shown to be associated with an increased risk of cerebral palsy (26). The results of the present study revealed a strong statistical association between maternal vascular malperfusion lesions and birth asphyxia (P=0.01), both moderate and severe.

The presence of vascular lesions increases the vulnerability of the fetus during labor, leading to hypoxic lesions at birth. Furthermore, these results underscore the presence of a persistent thrombotic mechanism occurring inside the fetal and placental blood vessels, which demonstrate a close interconnection. There is a positive association between hypoxia and the occurrence of clots inside the fetoplacental circulation. The findings of the present study align with those of Redline (22), who emphasized a statistically significant correlation between prenatal vascular malperfusion and hypoxic-ischemic encephalopathy.

The association between villous destruction, caused by chronic villitis of unknown etiology (P=0.01) or delayed villous maturation (P=0.01), and mild forms of the disease is not unexpected. This is due to the fact that these mechanisms contribute to the gradual development of lesions by activating stress-adaptive mechanisms in the fetus before birth. Consequently, the infant presents a reduced level of hypoxia and a better response at birth.

In contrast to the study by Wintermark *et al* (9), who reported an association between chorioamnionitis and brain injury, the results of the present study did not show any such association. This can be explained by the good protective function of the brain in acute injuries. Additionally, Strunk *et al* (27) found that the risk of late-onset sepsis for infants exposed to histological chorioamnionitis decreased. Although the present study included newborns with gestational ages <32 weeks, it is notable that the neonates were at term.

Previous research has established a connection between placental abruption and cerebral palsy (28). Although the diagnosis of placental abruption is primarily clinical, a histopathological examination has revealed the presence of retroplacental hematoma. Following the findings of Bingham *et al* (14), the appearance of retroplacental hematoma did not demonstrate a statistically significant association with the occurrence of ischemic hypoxic syndrome at birth. In addition, the present study did not reveal a significant association between the two factors.

Recent research, in accordance with the latest ISUOG guidelines, highlights the significance of evaluating the placenta and umbilical cord during the ultrasound examination in the third trimester of pregnancy (29). Therefore, the data collected regarding the position of the placenta and its specific type (such as previa or accreta) can determine the mode of delivery and the level of urgency in finishing the pregnancy, subsequently reducing the rate of perinatal mortality.

Furthermore, placenta previa increases the risk of umbilical cord abnormalities, such as velamentous cord insertion, marginal insertion and vasa previa. These conditions can be detected through an ultrasound examination during the third trimester of pregnancy. The umbilical cord abnormalities observed in the present study were associated with neonatal hypoxia (P=0.01) (30).

The present study stands out from the vast literature as, contrary to the numerous studies that have been published in the past, the particularity of the present study lies in the clear diagnostic criteria for perinatal asphyxia and a standardized classification of placental lesions. A notable strength of the present study is that we were able to identify placental lesions in a group of patients who had a clear diagnosis of birth asphyxia, as well as that we were able to categorize placental lesions according to Amsterdam criteria and that we identified a large number of lesions on the examined placenta. The limitations of the present study are linked to the relatively limited number of patients included, the unblinded nature of the single pathologist responsible for the placental examination, and the absence of brain imaging for newborns with hypoxic-ischemic syndrome.

In conclusion, the present study demonstrates that birth asphyxia is associated with micro- and macroscopic placental lesions, as well as abnormalities of the umbilical cord. In addition, the weight of the placenta tends to be greater in newborns with hypoxia at birth. Furthermore, the placental histopathological examination illustrating changes in both maternal and fetal vascular malperfusion is associated with the presence of hypoxic syndrome at birth.

Moreover, the categorization of patients based on the manifestation of the disease enabled us to emphasize that the less severe versions are linked to the existence of persistent villous inflammation and the postponement of delayed villous maturation. Furthermore, it has been observed that the presence of maternal and fetal vascular malperfusion-type lesions is related to moderate and severe types of hypoxic lesions.

## Figures and Tables

**Table I:** Patients characteristics.

Characteristic	Birth asphyxia (n=62)	Control (n=69)	P-value
Gestational age (weeks)	37.83±1.4	36.58±3.1	NS
Birth weight (g)	3,030.95±602.62	2,743.33±891.20	0.02
Cesarean section, n (%)			0.01
Yes	25 (40.3%)	12 (17.4%)	
No	37 (59.7%)	57 (82.5%)	
Median ventilation duration (days)	3.90±3.03	2.57±2.76	0.01
Length of hospital stay (days)	17.26±11.7	13.38±7.2	0.03
Male sex, n (%)			0.03
Yes	22 (35.5%)	14 (20.3%)	
No	40 (64.5%)	55 (79.7%)	
CTG abnormalities, n (%)			0.01
Yes	51 (82.3%)	27 (39.1%)	
No	11 (17. 7%)	42 (60.9%)	
Tachycardia, n (%)			0.01
Yes	18 (29.0%)	7 (10.1%)	
No	44 (71.0%)	62 (89.9%)	
Bradycardia, n (%)			0.01
Yes	23 (37.1%)	12 (17.4%)	
No	39 (62.9%)	57 (82.5%)	
Lower variability, n (%)			NS
Yes	10 (16.1%)	8 (11.6%)	
No	52 (83.9%)	61 (88.4%)	
Complicated pregnancies, n (%)			0.01
Yes	56 (90.3%)	36 (52.2%)	
No	6 (9.7%)	33 (47.8%)	
Pregnancy-induced hypertension, n (%)			0.01
Yes	18 (29.0%)	7 (10.1%)	
No	44 (71.0%)	62 (89.9%)	
Gestational diabetes, n (%)			NS
Yes	16 (25.8%)	17 (24.6%)	
No	46 (74.2%)	52 (75.4%)	
Preeclampsia, n (%)			0.02
Yes	11 (17.7%)	3 (4.3%)	
No	51 (82.3%)	66 (95.7%)	
Pregnancy cholestasis, n (%)			NS
Yes	11 (17.7%)	9 (13.0%)	
No	51 (82.3%)	60 (87.0%)	
Under psychiatric treatment, n (%)			NS
Yes	4 (6.4%)	1(1.4%)	
No	58 (93.6%)	68 (98.6%)	
Fetal growth restriction, n (%)			NS
Yes	21 (33.9%)	16 (23.2%)	
No	41 (66.1%)	53 (76.8%)	
APGAR at 5 min	4.92±1.5	5.94±1.42	0.01
Umbilical cord pH	7.05±0.09	7.23±0.07	0.01
Umbilical cord pCO_2_ (mmHg)	64.2±0.07	48.5±0.05	0.01
Umbilical cord base deficit (mmol/l)	-13.4±0.04	-5.0±0.02	0.01
pH at 1 h of life	7.12±0.07	7.27±0.02	0.01
pCO_2_ (mmH) at 1 h of life	56.5±0.04	40.4±0.03	0.01
Base deficit (mmol/l) at 1 h of life	-8.2±0.03	-2.3±0.06	0.02
SARNAT 1, percentage	23.5%	0	
SARNAT 2, percentage	26.5%	0	
SARNAT 3, percentage	13.6%	0	

CTG, cardiotocography; g, grams; NS, not significant.

**Table II:** Placental macroscopic examination data and umbilical cord abnormalities.

Parameter	Birth asphyxia (n=62)	Control (n=69)	P-value
Placental weight (g)	453.62±87.58	405.52±138.43	0.01
Umbilical knot, n (%)			0.01
Yes	10 (16.1%)	2 (2.9%)	
No	52 (83.9%)	67 (97.1%)	
Unique umbilical artery, n (%)			0.01
Yes	23 (37.1%)	12 (17.4%)	
No	39 (62.9%)	57 (82.6%)	
Insertion anomalies of the umbilical cord, n (%)			NS
Yes	18 (29.0%)	24 (34.8%)	
No	44 (71.0%)	45 (65.2%)	
Hypercoiled, n (%)			NS
Yes	4 (6.5%)	7 (10.1%)	
No	58 (93.5%)	62 (89.9%)	
Hypocoiled, n (%)			NS
Yes	2 (3.2%)	5 (7.2%)	
No	60 (96.8%)	62 (89.8%)	
Normal, n (%)			0.01
Yes	5 (8.1%)	19 (27.5%)	
No	57 (91.9%)	50 (72.5%)	

g, grams; NS, not significant.

**Table III:** Placental microscopic lesions.

Placental lesions	Birth asphyxia (n=62) (%)	Control (n=69) (%)	P-value
MVM, n (%)			0.01
Yes	15 (24.2)	4 (5.8)	
No	47 (75.8)	65 (94.2)	
FVM, n (%)			0.01
Yes	11 (17.7)	2 (2.9)	
No	51 (82.3)	67 (97.1)	
VUE, n (%)			0.03
Yes	10 (16.1)	3 (4.3)	
No	52 (83.9)	66 (65.7)	
Delayed villous maturation, n (%)			0.03
Yes	12 (19.4)	4 (5.8)	
No	50 (80.6)	65 (94.2)	
Chorioamnionitis, n (%)			NS
Yes	5 (8.1)	9 (13.0)	
No	57 (91.9)	60 (87.0)	
Abruption, n (%)			0.05
Yes	6 (9.7)	1 (1.4)	
No	56 (81.3)	68 (98.6)	
No lesions, n (%)			0.01
Yes	3 (4.8)	47 (68.1)	
No	59 (95.2)	22 (31.9)	

MVM, maternal vascular malperfusion; FVM, fetal vascular malperfusion; VUE, chronic villitis of unknown etiology; NS, not significant.

**Table IV:** Placental microscopic lesions in those with mild, moderate and severe asphyxia, and the controls.

A, Those with mild asphyxia vs. the controls			
Placental lesions	Mild asphyxia (n=23) (%)	Control (n=69) (%)	P-value
MVM, n (%)			NS
Yes	1 (4.3)	4 (5.8)	
No	22 (95.7)	65 (94.2)	
FVM, n (%)			NS
Yes	0 (0)	2 (2.9)	
No	23(100)	67 (97.1)	
VUE, n (%)			0.01
Yes	7 (30.4)	3 (4.3)	
No	16 (69.6)	66 (65.7)	
Delayed villous maturation, n (%)			0.01
Yes	10 (43.5)	4 (5.8)	
No	13 (56.5)	65 (94.2)	
Chorioamnionitis, n (%)			
Yes	1 (4.3)	9 (13.0)	
No	22 (95.7)	60 (87.0)	
Abruption, n (%)			NS
Yes	1 (4.3)	1 (1.4)	
No	22 (95.7)	68 (98.6)	
Normal, n (%)			0.01
Yes	3 (13.0)	47 (68.1)	
No	20 (87.0)	22 (31.9)	
B, Those with moderate asphyxia vs. the controls			
Placental lesions	Moderate asphyxia (n=16) (%)	Control (n=69) (%)	P-value
MVM, n (%)			NS
Yes	3 (18.8)	4 (5.8)	
No	13 (81.2)	65 (94.2)	
FVM, n (%)			0.01
Yes	4 (25.0)	2 (2.9)	
No	12(75)	67 (97.1)	
VUE, n (%)			NS
Yes	2 (12.5)	3 (4.3)	
No	14 (87.5)	66 (65.7)	
Delayed villous maturation, n (%)			NS
Yes	2 (12.5)	4 (5.8)	
No	14 (87.5)	65 (94.2)	
Chorioamnionitis, n (%)			NS
Yes	2 (12.5)	9 (13.0)	
No	14 (87.5)	60 (87.0)	
Abruption, n (%)			0.02
Yes	3 (18.8)	1 (1.4)	
No	13 (81.2)	68 (98.6)	
Normal, n (%)			0.01
Yes	0 (0)	47 (68.1)	
No	16(100)	22 (31.9)	
C, Those with severe asphyxia vs. the controls			
Placental lesions	Severe asphyxia (n=23) (%)	Control (n=69) (%)	P-value
MVM, n (%)			0.01
Yes	11 (47.8)	4 (5.8)	
No	12 (52.2)	65 (94.2)	
FVM, n (%)			0.01
Yes	7 (30.4)	2 (2.9)	
No	16 (69.6)	67 (97.1)	
VUE, n (%)			NS
Yes	1 (4.3)	3 (4.3)	
No	22 (95.7)	66 (65.7)	
Delayed villous maturation, n (%)			NS
Yes	0 (0)	4 (5.8)	
No	23(100)	65 (94.2)	
Chorioamnionitis, n (%)			NS
Yes	2 (8.7)	9 (13.0)	
No	21 (91.3)	60 (87.0)	
Abruption, n (%)			NS
Yes	2 (8.7)	1 (1.4)	
No	21 (91.3)	68 (98.6)	
Normal, n (%)			0.01
Yes	0 (0)	47 (68.1)	
No	23(100)	22 (31.9)	

MVM, maternal vascular malperfusion; FVM, fetal vascular malperfusion; VUE, chronic villitis of unknown etiology; NS, not significant.

## Data Availability

The datasets used and/or analyzed during the current study are available from the corresponding author on reasonable request.
